# Idiopathic intracranial hypertension with juvenile idiopathic arthritis‐associated uveitis: A case report

**DOI:** 10.1002/ccr3.4281

**Published:** 2021-06-23

**Authors:** Asaad Alkoht, Huda Alhariry, Ibrahem Hanafi, Majed Aboud

**Affiliations:** ^1^ Faculty of Medicine Division of Rheumatology Department of Internal Medicine Damascus University Damascus Syria; ^2^ Faculty of Medicine Division of Neurology Department of Internal Medicine Damascus University Damascus Syria

**Keywords:** idiopathic intracranial hypertension, juvenile idiopathic arthritis, pseudotumor cerebri, uveitis

## Abstract

A 14‐year‐old girl with juvenile idiopathic arthritis (JIA)‐associated uveitis who also had optic disc edema, was later diagnosed with Idiopathic intracranial hypertension (IIH). To our knowledge, this is the fifth case of the coexistence of uveitis and IIH among children, and the only one with no obvious risk factors for IIH.

## Graphical abstract

In JIA‐associated uveitis with bilateral disk edema, IIH should be kept as a differential diagnosis. Especially if disk edema does not resolve after resolution of the ocular inflammation.

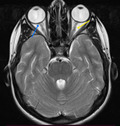

## 1 INTRODUCTION

Idiopathic intracranial hypertension (IIH), also called pseudotumor cerebri, is a disorder of elevated pressure of the cerebrospinal fluid (CSF) with no evident cause (ie, normal neuroimaging and CSF tests).^1^, ^2^ It is a rare condition with an incidence of 0.1‐0.9 per 100 000 population, and it predominantly affects obese women of childbearing age with a smaller percentage of cases occurring in children.^3^, ^4^, ^5^, ^6^

Intracranial hypertension commonly presents with headache, transient visual obscurations, and pulsatile tinnitus,^1^ while the most frequent signs are papilledema, visual field defect, and sixth nerve palsy.^7^, ^8^ IIH is associated with many systemic illnesses such as Addison disease, hypoparathyroidism, and anemia, as well as, medications like corticosteroids, vitamin A (V.A), and tetracyclines.^9^, ^10^, ^11^, ^12^, ^13^, ^14^, ^15^, ^16^ Besides that, IIH risk factors include obesity and family history.^7^, ^8^, ^9^ To our best knowledge, the association of IIH with uveitis has been reported in only four children so far.^17^, ^18^, ^19^ In this report, we discuss the case of a girl diagnosed with juvenile idiopathic arthritis (JIA)‐associated uveitis who developed IIH without any apparent risk factor.

## 2 CASE PRESENTATION

A 14‐year‐old girl presented to our hospital with a 2‐day complaint of bilateral painful red eyes with mild blurred vision preceded by a month of persistent low back pain that did not improve on rest. She also had a 2‐year history of recurrent inflammatory arthritis and half‐hour morning stiffness with no obvious swelling for which she had been treated intermittently with nonsteroidal anti‐inflammatory drugs. Moreover, the joint pain had become continuous and more intense 10 days before presenting to our center with fever peaks several times a day. Without a confirmed diagnosis, she had been prescribed prednisolone 20 mg daily for just 3 days, followed by a treatment regimen for brucellosis (gentamicin, doxycycline, and rifampicin) for 5 days without improvement. She had no relevant past medical, travel, or family history.

**FIGURE 1:**
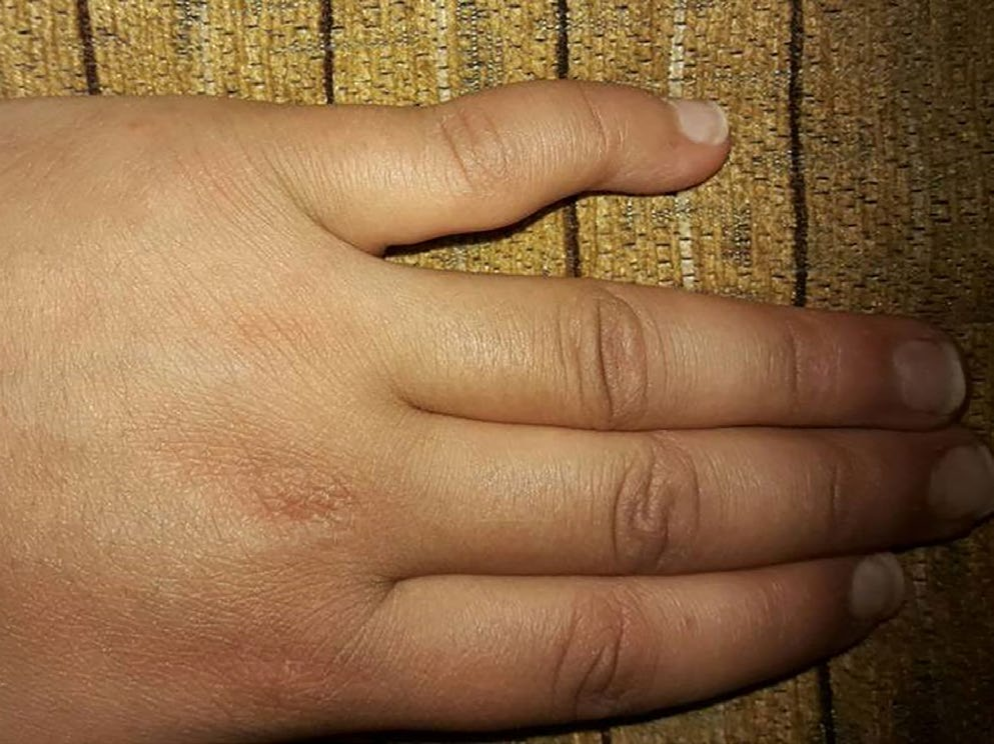
The left‐hand shows mild swan‐neck deformity in the fifth finger

On physical examination, her blood pressure was 120/80 mm Hg, pulse was 105 beats per minute and regular, respiratory rate was 23 cycles per minute, the temperature was 38.5°C, and body mass index was 20 kg/m^2^. She had red eyes, tenderness in the right elbow and both ankles, as well as a swan‐neck deformity in the fifth finger in both hands (Figure 1). The rest of the physical examination was normal.

On ophthalmic examination, her distance visual acuity without correction was 20/20 in both eyes. Slit‐lamp examination revealed bilateral precipitates on the endothelium of cornea and the anterior surface of the lens, cells (3+) in both anterior chambers with few cells in the anterior vitreous, which demonstrated anterior uveitis. She also had grade 1 optic disk edema bilaterally. The macula, peripheral retina, and vessels in each eye were normal. Intraocular Pressure (IOP) was normal.

Laboratory investigations revealed a blood leukocyte count of 12.4 K/mm^3^ with 85% neutrophils, a hemoglobin concentration of 12.2 g/dL, and a platelet count of 340 K/mm^3^. Erythrocyte sedimentation rate (ESR) was 50 mm at the end of the first hour and C‐reactive protein (CRP) was 4.6 mg/L. Liver function tests, creatinine, urea, urinalysis, and microscopy were within normal limits. Antinuclear antibody (ANA), rheumatoid factor (RF), HIV antibodies, VDRL, HBsAg, anti‐HCV, Brucella IgG Ab, Brucella IgM Ab, and blood culture were all negative. Tuberculin test was negative after 48‐72 hours. X‐ray of the hands showed malalignment with joint space narrowing in the proximal interphalangeal joints of the fifth fingers in both hands. Chest, pelvis, and lumbosacral spine X‐ray images were within normal limits. The echocardiogram was normal with no evidence of endocarditis.

**FIGURE 2:**
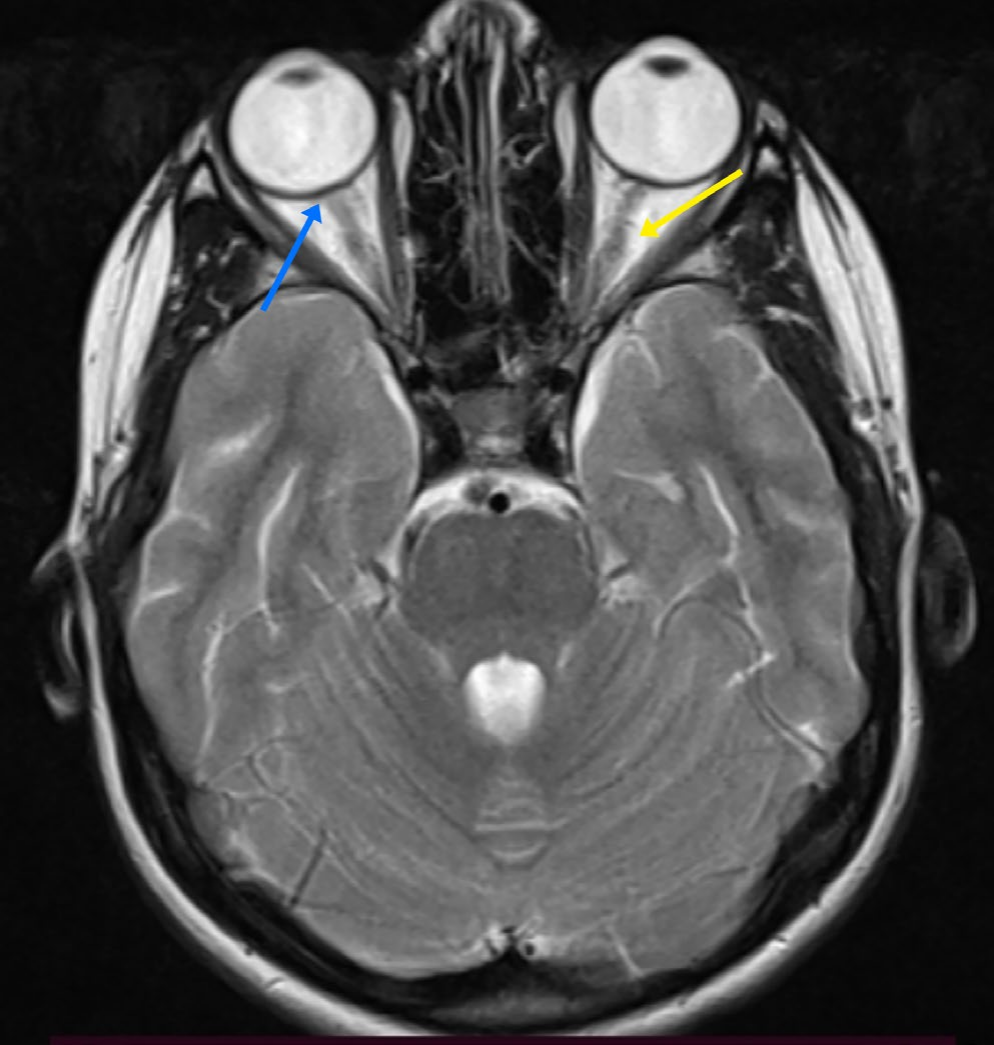
Brain magnetic resonance imaging showing mild optic nerve tortuosity (yellow arrow) and posterior globe flattening (blue arrow)

**FIGURE 3:**
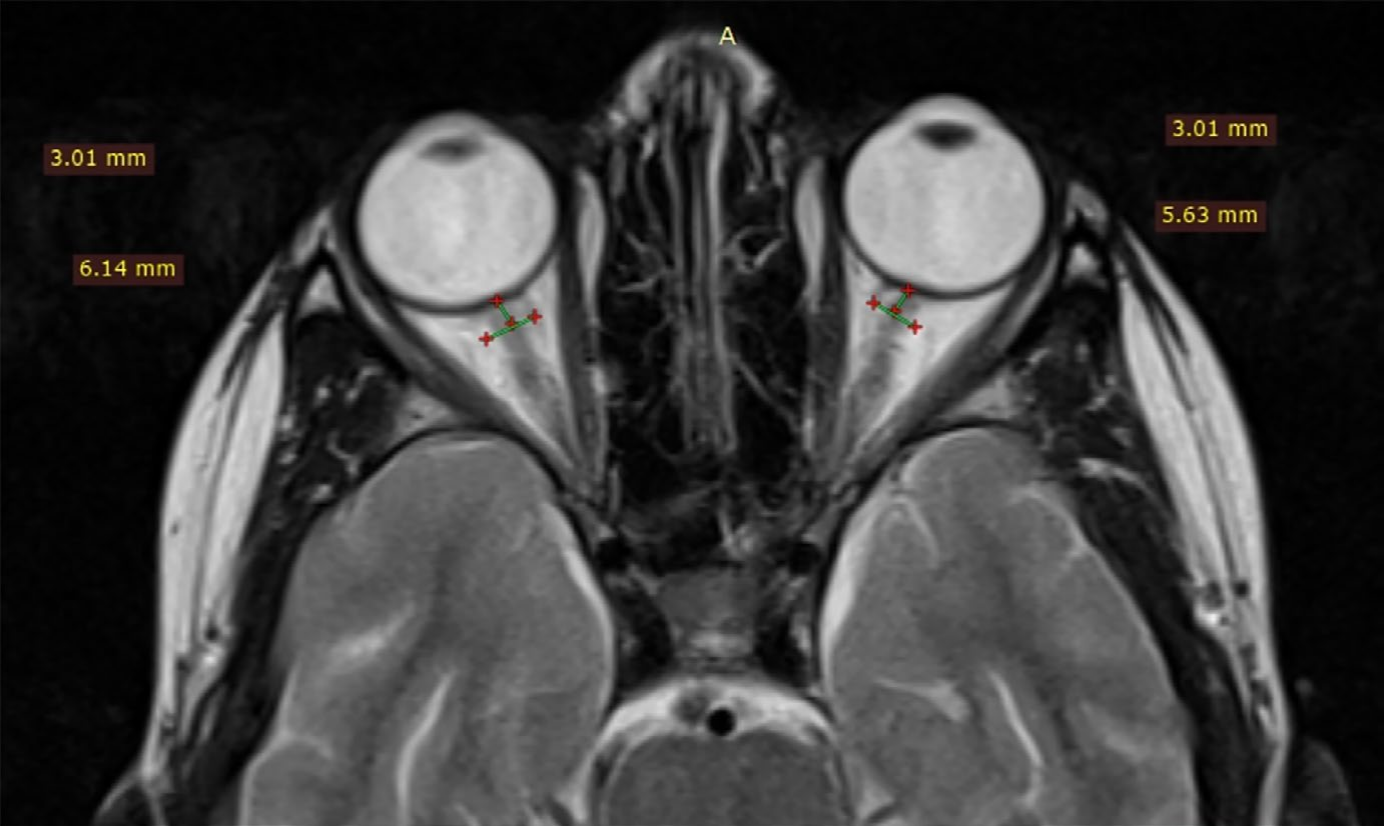
Brain magnetic resonance imaging showing prominent subarachnoid space around the optic nerves

After ruling out infectious causes, malignancies, and other systemic autoimmune diseases, the patient was diagnosed with enthesitis‐related JIA based on the International League of Associations for Rheumatology (ILAR) classification criteria.^20^ She was treated with prednisolone 0.5 mg/kg/day, which led to an improvement in fever, eyes redness, and articular manifestations within 3 days. On discharge, we added methotrexate 10 mg once a week. Seven days later, she returned for follow‐up with a new complaint of generalized, persistent, tension headache that improved partially on analgesics, accompanied by blurred vision. Ophthalmic examination showed normal visual acuity with grade 3 optic disk edema in both eyes with no flare in the anterior chamber and vitreous. Vital signs were all normal. Brain computed tomography (CT) was normal and lumbar puncture disclosed a slightly increased opening pressure of 300 mm H_2_O. CSF analysis revealed no cells, a CSF protein of 40 mg/dL (normal up to 45 mg/dL), and a CSF glucose of 56 mg/dL with serum glucose of 79 mg/dL. Total blood leukocyte count was 10.4 K/mm^3^ with a differential count of neutrophil 86%. ESR was 20 mm at the end of the first hour. All other laboratory studies were normal. Magnetic resonance imaging (MRI) of the brain showed mild optic nerve tortuosity, posterior globe flattening (Figure 2), and prominent subarachnoid space around the optic nerves (Figure 3), while magnetic resonance venography (MRV) was normal. Based on revised diagnostic criteria, the patient was diagnosed with IIH ^2^ and was put on acetazolamide 500 mg/day then the dose increased up to 750 mg daily. Three weeks later, the papilledema and the headache had resolved and acetazolamide and prednisolone were tapered off. After 2‐year of follow‐up, she was still doing well on a 2.5 mg methotrexate maintenance dose weekly.

## 3 DISCUSSION

We presented the case of a girl with arthritis, uveitis, and optic disk edema, who was later diagnosed with IIH. Uveitis is an intraocular inflammation that can be idiopathic or associated with underlying systemic diseases. Therefore, thorough investigations were done leading to the diagnosis of JIA‐associated uveitis after excluding other systemic etiologies. Since optic disk edema may be found in patients with uveitis and resolves after the treatment of uveitis,^21^, ^22^ we initially opted to only monitor it. However, 18 days later the patient developed a headache and she was diagnosed with IIH which should be the cause of optic disk edema. Even though IIH is typically symptomatic in her age group, she had an atypical presentation at first similar to younger children who are frequently asymptomatic.^18^, ^23^ Subsequently, the absence of headache and other symptoms apparently was not enough to exclude the diagnosis of IIH, especially when optic disk edema did not improve after the treatment of uveitis.

Our patient had two presumed risk factors for IIH. One of them is corticosteroids which were given for three consecutive days, 26 days before the diagnosis of IIH, as well as for 14 days as a treatment for uveitis without tapering‐off. However, the risk of IIH in the case of corticosteroids is seen when they are tapered after long‐term use, which does not apply to our case.^6^, ^14^ The other risk factor is doxycycline, one of the tetracyclines that have been associated with IIH in several cases, with a notice that in these reports, tetracycline was often combined with other assumed risk factors.^14^, ^15^, ^16^ IIH frequently develops within a few weeks to months after treatment initiation and sometimes cessation of the drug is enough for recovery.^14^, ^15^, ^16^ Our patient was treated with doxycycline for just 5 days before the first admission and 23 days before developing the headache, therefore, doxycycline cannot also be considered associated with IIH in our case.

To our knowledge, four juvenile cases were reported to date as having uveitis and IIH (Table 1‐A); three of them had anterior uveitis similar to our case and one had panuveitis. Only one of the cases had JIA‐associated uveitis similar to our report.

**TABLE 1:** Pediatric cases of Idiopathic Intracranial Hypertension with Juvenile Idiopathic Arthritis and/or Uveitis

	Cases	Age/Sex	IIH	Uveitis	JIA	Associated conditions	Associated drugs	Treatment^a^
Section A: With uveitis	Margalit^17^	Girl 11‐y	Present symptomatic	Panuveitis	—	Weight gain	—	Acetazolamide, weight reduction
	Buscher^19^	Boy 11‐y	Present asymptomatic	Anterior uveitis	—	Weight gain	Cyclosporine	Acetazolamide, prednisone, MMF^b^
	Curragh first case^18^	Girl 8‐y	Present asymptomatic	Anterior uveitis	Oligoarticular JIA	—	Steroids	Furosemide^c^
	Curragh second case^18^	Boy 5‐y	Present asymptomatic	Anterior uveitis	—	—	Steroids	Acetazolamide
Section B: Without uveitis	Burstzyn^24^	Child unknown	Present symptomatic	—	Systemic JIA	—	Steroids	Lumbar puncture, acetazolamide, ONSF^d^
	Bhettay^25^	Child unknown	Present unknown	—	Undefined JIA	—	V.A	V.A cessation
	Wanigasinghe^23^	Boy 5‐y	Present symptomatic	—	Systemic JIA	—	Steroids ^e^, V.A	Acetazolamide, furosemide
	Incecik^26^	Girl 9‐y	Present symptomatic	—	Undefined JIA	—	Methotrexate	Acetazolamide, methotrexate cessation
Section C:	Our case	Girl 14‐y	Present symptomatic^f^	Anterior uveitis	Enthesitis‐related JIA	—	—	Acetazolamide

^a^ Treatment of IIH and associated disease.

^b^ Mycophenolate mofetil.

^c^ Acetazolamide was commenced but not tolerated.

^d^ Unilateral optic nerve sheath fenestration.

^e^ Without withdrawal.

^f^ Asymptomatic at first.

A further search revealed four other cases of reported IIH complicated with JIA without uveitis (Table 1‐B). One of them was a girl who had been treated with methotrexate without steroid for 1 year before being diagnosed with IIH. Methotrexate was considered a suspected cause, so it was stopped and the patient was given acetazolamide, leading to the improvement in symptoms a week later. On the contrary, our patient was kept on methotrexate after the diagnosis of IIH and improved nevertheless.

All former cases had IIH with possible predisposing drugs and/or conditions in contrast to our patient who did not have any certain ones (Table 1). As treatment of pediatric patients with IIH is empiric due to the lack of enough clinical trials, most cases, including ours, were treated with acetazolamide in addition to opposing predisposing factors when present.

This case report has a potential limitation. There is a possibility that IIH and uveitis were unrelated entities. However, IIH should be suspected in cases of uveitis complicated with optic disk edema, as prompt diagnosis and treatment can prevent vision loss.

## CONFLICT OF INTEREST

None declared.

## AUTHOR CONTRIBUTIONS

AA: Did the literature search and drafted the discussion; HA: Drafted the case presentation; IH: Drafted the introduction and edited the paper for submission; MA and all authors reviewed the article and approved the last version of it.

## CONSENT FOR PUBLICATION

Informed consent has been obtained.

## Data Availability

The medical records of our patient are saved in the archive in our center.
